# Microbial exopolysaccharides as bioactive modulators of intestinal health: a systematic review

**DOI:** 10.3389/fnut.2026.1885879

**Published:** 2026-09-07

**Authors:** Chloe J. McGovern, Elad Tako

**Affiliations:** Department of Food Science and Technology, Cornell University, Ithaca, NY, United States

**Keywords:** exopolysaccharides, gut microbiome, intestinal epithelium, intestinal health, microbial metabolites

## Abstract

Microbial exopolysaccharides (EPS) are secondary metabolites produced and secreted by microorganisms during fermentation. Their structure and physicochemical properties depend on the microbial strain and growth conditions, making them highly versatile for application in the food industry. Current investigations have expanded to include their use as a bioactive compound, providing health benefits to the host without the introduction of live microorganisms, as in the case with probiotics. This systematic review analyzed 28 *in vivo* studies to assess the effects of EPS on intestinal health. Evidence suggests that EPS modulates the gut microbiome, enhances intestinal epithelium integrity, alters inflammatory biomarkers, affects colonic morphology, and increases short-chain fatty acid (SCFA) production. Overall, this systematic review primarily focuses on colonic health, with limited consideration of the role of EPS during small intestinal digestion. The findings support the potential of microbial exopolysaccharides as bioactive macromolecules that may improve intestinal health. This systematic review has been registered with the International Prospective Register of Systematic Reviews database (PROSPERO) (ID: CRD420261292367).

## 1 Introduction

Probiotics have demonstrated beneficial health effects both *in vitro* and *in vivo*, particularly in mitigating inflammation associated with inflammatory bowel disease (1), promoting the expression of tight junction proteins (2, 3), and supporting intestinal barrier integrity, including mucin production (4–6). However, probiotics are not always suitable for severely compromised gut barriers. When the intestinal epithelium is damaged, as seen in ulcerative colitis and Crohn’s disease, bacteria may translocate across the intestinal barrier and enter the bloodstream (7–9). Retrospective analysis of intensive care unit (ICU) patient data has shown that administered *Lactobacillus* strains can translocate into the bloodstream, resulting in bacteremia (10). Similar studies have suggested that *Lactobacillus-*associated bacteremia may be linked to probiotic supplementation, highlighting the need for caution in the use of probiotics in individuals with compromised gut barriers (11, 12).

Microbial metabolites are a proposed therapeutic for gut dysbiosis and inflammatory bowel disease that exhibit bioactive properties associated with probiotics without the introduction of live bacteria (13–15). Exopolysaccharides (EPS) are high-molecular-weight carbohydrate polymers secreted by or anchored to the surface of many probiotic and commensal bacterial taxa (16, 17). EPS have been explored as a functional ingredient in the food industry due to physicochemical and bioactive potential (18). EPS as an ingredient has been shown in modulating texture and viscosity in dairy food products (19–22). Specifically, xanthan gum is a microbial exopolysaccharide that is commonly used by industry as a food additive (23, 24). With a direct application in the food industry, EPS are valuable metabolites that can be leveraged as a functional ingredient with bioactive and physicochemical functionality (25).

This systematic review provides the first comprehensive analysis of studies investigating the effects of microbial exopolysaccharides on intestinal health biomarkers, including histological measurements, epithelial integrity, and gut microbiome profiles in both healthy and diseased conditions. Specifically, this systematic review aims to evaluate whether microbial exopolysaccharides improve intestinal health biomarkers in animal models. We aimed to conduct comprehensive search and analysis of *in vivo* animal studies investigating the effects of microbial exopolysaccharides on intestinal health outcomes, including inflammation, epithelial barrier integrity, intestinal morphology, microbiota composition, and short-chain fatty acid (SCFA) profiles.

## 2 Methods

### 2.1 Protocol and registration

This systematic review is intended to critically evaluate science addressing the question, “What is the effect of exopolysaccharides on intestinal health?” The review protocol follows the Preferred Reporting Items for Systematic Reviews and Meta-Analyses (PRISMA) guidelines (26). The protocol was submitted and published to the International Prospective Register of Systematic Reviews database (PROSPERO) under the register ID of CRD420261292367.

### 2.2 Eligibility criteria

The PICOS criteria for inclusion and exclusion of manuscripts were used in this systematic review (Table 1). In the population parameter, *in vivo* manuscripts were included in the analysis and assay-driven bioactivity analysis, characterization, *in vitro*, and clinical trials were excluded. All the manuscripts in this study the effect of microbial exopolysaccharides isolated effect on intestinal health with appropriate controls. Manuscripts that do not include proper controls or mixed exopolysaccharide effects with additional compounds or foods were excluded. To be included, studies had to have evaluated at least one primary outcome related to intestinal health, specifically relating to the intestinal microbiome compositions, SCFA production and profile, intestinal morphology, inflammation markers, or gene expression. Additionally, review manuscripts, clinical manuscripts, book chapters, conference abstracts, and manuscripts published in languages other than English were excluded.

**Table 1:** PICOS criteria for inclusion or exclusion of manuscripts.

Parameter	Inclusion criteria	Exclusion criteria
Population	*In vivo* manuscripts	Clinical trials and *in vitro* manuscripts
Intervention or Exposure	Microbial exopolysaccharides crude or pure (specifically as metabolic metabolite and food ingredient)	Symbiotics or postbiotics or combined with other compounds
Comparison	Control	No control
Outcome	Intestinal health (morphology, gut microbiome composition, SCFA profile, inflammation, oxidative stress, gut barrier function, brush border membrane)	No intestinal health parameters measured
Study Design	Experimental manuscripts	Review, book chapter, theses, dissertations, clinical manuscripts, manuscripts in other languages than English

### 2.3 Search strategy

Literature for this review was curated from four databases: PubMed,^1^ SCOPUS,^2^ Web of Science,^3^ and Food Science and Technology Abstracts (FSTA).^4^ A combination of key terms was used to find manuscripts that investigated the effect of microbial exopolysaccharides on intestinal functionality. Database-specific search strings, with syntax adapted to each platform are provided in Supplementary Table S1. Broad key terms to describe microbial exopolysaccharides were used to ensure any potential nomenclature were not overlooked in during compilation. Date of publication was not used as restriction criterion.

### 2.4 Selection of manuscripts and data extraction

Manuscript screening, full-text review, and data extraction were conducted by a single reviewer. Manuscripts were selected after reading the titles and abstracts. Manuscripts with unclear eligibility were read in full to resolve the uncertainty. Manuscripts were managed by Rayyan software (27). Manuscripts were selected if they investigated the effect of microbial exopolysaccharides on intestinal health using *in vivo* models. The following information was extracted from the selected manuscripts: author, year, country, animal model, study design, primary outcomes (parameters of intestinal health), and secondary outcomes (effects on other tissue). A quantitative meta-analysis was not performed due to substantial heterogeneity in EPS microbial source, structural characterization, dosing, and outcome measures across the included studies; a qualitative narrative synthesis, based on statistically significant (*p* < 0.05) directional changes reported in each manuscript, was therefore used to summarize outcomes.

### 2.5 Risk of bias

The selected manuscripts in this systematic review were analyzed using the Systematic Review Centre for Laboratory Animal Experimentation (SYRCLE) Risk of Bias (RoB) method for animal manuscripts (28). The assessment followed 10 domains that evaluate bias within the manuscripts. The domains were sequence generation, baseline characteristics, allocation concealment, random housing, blinding (performance bias), random outcome assessment, blinding (detection bias), incomplete outcome data, selective outcome reporting, and other sources of bias. Questions were answered with a yes, unclear, or no with green, yellow, and red colors and corresponding symbols of a plus sign (+), question mark (?), or minus sign (−), respectively. The risk of bias assessment was summarized with a traffic light plot.

## 3 Results

### 3.1 Selection of manuscripts

**Figure 1:**
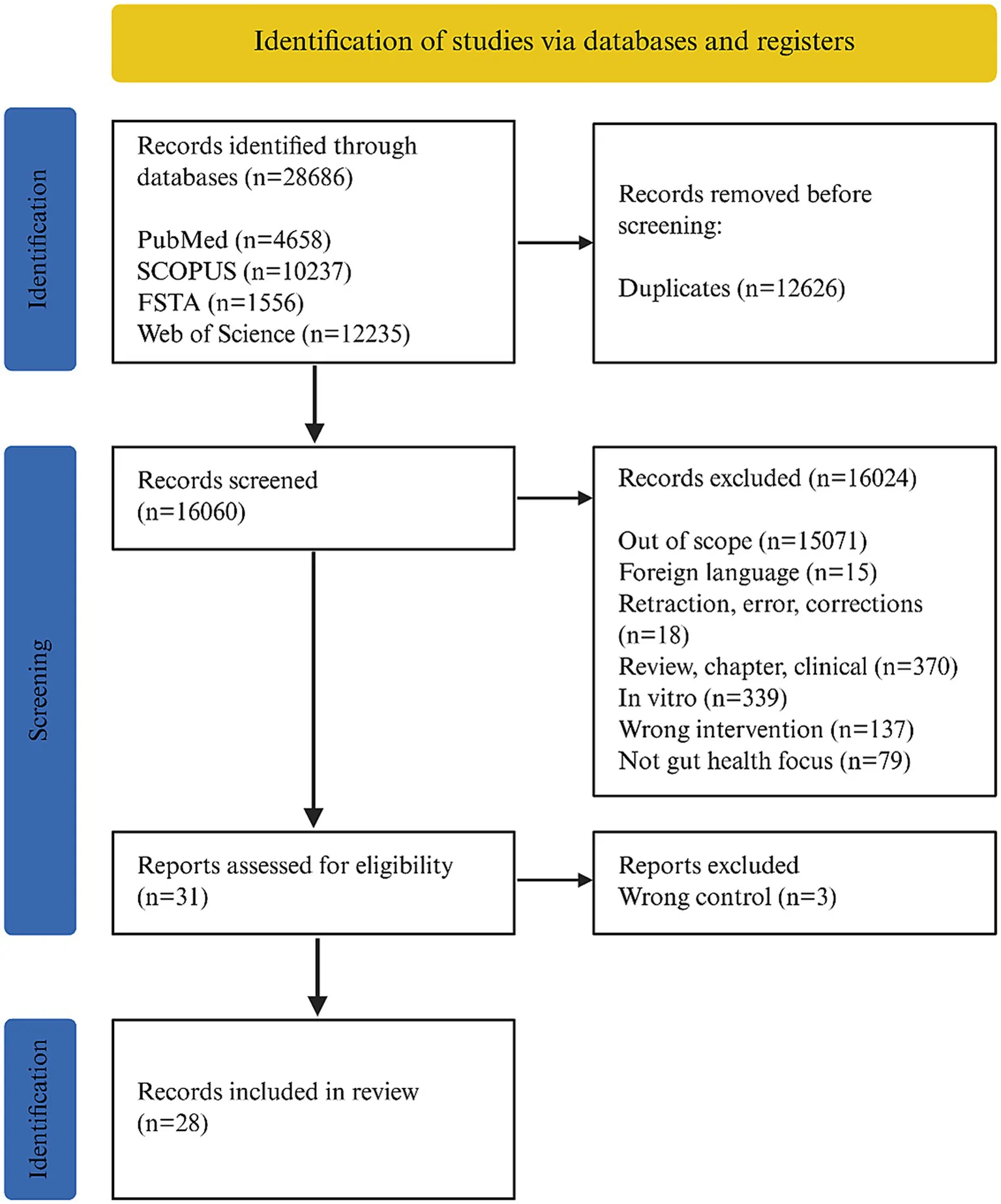
PRISMA flow diagram for manuscript inclusion.

There were 28,686 manuscripts pulled from the four databases (Figure 1). From there, 12,626 duplicate manuscripts were identified and removed in Rayyan by comparing title, abstract, authors, and DOI for exact matches. Following de-duplication, 16,060 manuscripts were screened by title and abstract. Due to the broad key terms and the intentional inclusion of “EPS” abbreviation, 15,071 manuscripts were deemed out of scope relating to just pre- or probiotics, aquaculture, sludge, oil spills, salinity, soil science, and electricity to name a few example topics. Manuscripts that were solely focused on genomics, fermentation optimization, characterization or physicochemical functionality of microbial exopolysaccharides were deemed out of scope. Additionally, manuscripts focused exclusively on health issues such as skin wounds, breast cancer, or oral/dental diseases were marked out of scope. From there, 15 manuscripts were not published in English and 18 were reported with errors, corrections, or redactions. Manuscripts within the relative scope were screened again (title and abstract) which resulted in 370 manuscripts being excluded for being some form of review, book, chapter, or clinical study, and 339 were *in vitro* models (digestion, fecal fermentation, or cell lines). Lastly, manuscripts that were incorrect treatment such as incorrectly identified exopolysaccharides were removed (*n* = 137) and manuscripts that did have the accurately reported microbial exopolysaccharides but did not measure intestinal health biomarkers were removed (*n* = 79). The resulting 31 manuscripts were read in depth, resulting in three manuscripts being removed from improper controls. This process resulted in 28 manuscripts that were deemed in the scope of this review.

### 3.2 Characterization of manuscripts included

The studies included in this systematic review were conducted across four countries. Of the 28 manuscripts in this analysis, 1 was reported being from Japan (29), 1 was from South Korea (30), 1 was conducted in Argentina (31), and 25 manuscripts were produced in China (32–56).

The *in vivo* models that were used to study microbial exopolysaccharide impact on intestinal health were zebrafish (1 of 28) (40) and mice (27 of 28) (29–39, 41–56). The manuscripts that used mice models were comprised of BALB/c mice (*n* = 8) (31, 33, 35, 38, 46, 51, 53, 56), C57BL/6 J (*n* = 9) (34, 36, 37, 43, 44, 48, 50, 52, 54), ICR (*n* = 1) (42), C57BL/6 J Jms Slc (*n* = 1) (29), Kunming (*n* = 1) (32), C57BL/6 N (*n* = 1) (47), and C57BL/6 (*n* = 6) which is an unspecified strain of mice (30, 39, 41, 45, 49, 55). Of the mice models, 1 did not report the sex of the mice used (51), 5 used female mice (31, 32, 38, 45, 46), and 21 used male mice (24, 25, 28–31, 34, 36–39, 42–45, 47–51). The age of the mice ranged: 3 weeks old (*n* = 1) (54), 4–6 weeks old (*n* = 1) (51), 4 weeks old (*n* = 1) (47), 6 weeks old (*n* = 7) (41, 43, 44, 48, 49, 52, 56), 6–8 weeks old (*n* = 6) (30, 31, 33, 38, 46, 50), 7 weeks old (*n* = 2) (29, 39), 7–8 weeks old (*n* = 1) (35), 8 weeks old (*n* = 4) (34, 36, 37, 55), and unknown age (*n* = 4) (32, 42, 45, 53). The manuscript reporting results found in the zebrafish model had a mix of adult (3 months of age) male and female fish (40).

### 3.3 Exopolysaccharide profile

All manuscripts that are included in this systematic review reported the microbial strain used to produce the EPS evaluated *in vivo*. Most of the studies derived EPS from bacteria (*n* = 25), with a majority originating from the *Lactobacillaceae* family (*n* = 17) (29, 30, 32, 37, 39–42, 44–47, 52–56) due to the 2020 reclassification of the *Lactobacillus* family (57), the *Bifidobacterium* genera (*n* = 3) (36, 38, 43), the *Streptococcus* genera (*n* = 1) (33), the *Bacillus* genera (*n* = 2) (34, 35), and the *Weissella* genera (*n* = 1) (50). The other 4 EPS manuscripts were extracted from fungi (*n* = 3) (48, 49, 51), and 1 study (31) extracted from a microbial consortium known to contain both prokaryotes and eukaryotes (kefir grains).

The majority of the manuscripts that were analyzed in this systematic review reported whether crude (*n* = 9) or pure (*n* = 17) extracts were used. The characteristics of EPS that are reported in the manuscript are reported in Table 2. There are 13 manuscripts with no reported information on EPS characteristics (29, 31, 36–39, 41, 42, 44, 45, 48, 51, 56). However, the remaining 15 manuscripts varied in information provided, from molecular weight to bond structure, using FT-IR and NMR. Additionally, chromatography was used for monosaccharide composition of investigated EPS (Table 2).

**Table 2:** Characteristics of microbial exopolysaccharides in selected manuscripts.

Author, Year, Country	Microbial source for EPS	Crude or purified	Molecular weight	Monosaccharide composition	Characterization technique(s)	Other structural notes
Zhao et al., 2025, (54) China	*Lactobacillus acidophilus* YL01 isolated from yogurt in the Hetao area in China	Pure	952 kDa	Rhamnose, arabinose, galactose, glucose, mannose, glucuronic acid (molar ratio 0.27:1.15:1.05:8.37:4.27:1)	HPLC; FT-IR	Classified as an acidic polysaccharide
Gao et al., 2025, (34) China	*Bacillus thuringiensis* IX-01 isolated from Sichuan’s Pixian bean paste	Pure	Not reported	GlcNAc, GaINAc, Glcp, Manp, Araf units (branched structure)	Previously published paper	Backbone: four 1,4-β-GlcNAc, four 1,4-β-GaINAc, two 1,4-α-Glcp; branches: T-β-Glcp, 1,4-β-Manp, 1,6-α-GaINAc, two 1,5-α-Araf (10–11 units)
Wang et al., 2025, (47) China	*Levilactobacillus brevis* M-10 isolated from fermented broomcorn millet sour porridge	Pure	~1,520 kDa	Mannose, glucose (9:1)	Not reported	Main chain: →3)-α-D-Manp-(1 → and 2)-α-D-Manp-(1→; branch: →4)-α-D-Glcp-(1 → linked at O-3 of main chain
Su et al., 2025, (44) China	*Lacticaseibacillus paracasei* JY062 isolated from Tibetan fermented dairy foods	Crude	Unknown	Unknown	Unknown	Unknown
Zhang et al., 2025, (53) China	*Lacticaseibacillus rhamnosus* LRa05	Crude	2.89 × 10^6^ Da	Mannose, glucose, galactose (25:70:5)	HPLC, GC–MS, UV-spec	Total carbohydrate 82.35%; protein 3.04%
Li et al., 2025, (30) South Korea	*Lactiplantibacillus plantarum* ZL1	Pure	169.6 kDa	Rhamnose 5.06%, galactose 25.79%, mannose 53.37%, glucose 10.93, <1% glucuronic acid	FT-IR; GC–MS; NMR	α-anomeric configuration; GC–MS sugar residues →2)-Rhap, Manp, Glcp, →3)-Glcp, →4)-Manp, →4)-Galp, →3,6)-Glcp, →2,6)-Manp
Li, H, et al., 2024b, (36) China	*Bifidobacterium longum subsp. longum* YS108R and *B. longum subsp. longum* C11A10B	Crude	Unknown	Unknown	Unknown	Unknown
Xie et al., 2024, (49) China	*Monascus purpureus* 40,269	Pure	56.4 kDa	Arabinose 0.87%, xylose 0.31%, mannose 37.52%, galactose 33.73%, glucose 27.57%	FT-IR; NMR	Fraction B used; yield 28.37%; total sugar content 90.38%; 2 linkage types identified by NMR
Li, H, et al., 2024a, (36) China	*Limosilactobacillus mucosae* CCFM1273	Crude	Unknown	Unknown	Unknown	Unknown
Chen et al., 2024, (32) China	*Lacticaseibacillus rhamnosus* ZFM216 isolated from fresh milk	Crude	19.9 kDa	Guluronic acid, mannuronic acid, mannose, ribose, rhamnose, galacturonic acid, glucose, xylose, galactose, arabinose (95.2:1:19.2:1.4:3.3:1.2:106.8:1.4:5.9:13.6)	Previously published paper	Total sugar 70.06%; protein 1.44%; uronic acid 17.74%
Zhang et al., 2024, (52) China	*Lactobacillus helveticus* 6 M3, *L. helveticus* DQHXNQ5M22, *L. plantarum* VJLHD4L1, and *L. plantarum* CCFM 1393	Crude	Two fractions: 100 kDa and 5 kDa	Glucose, galactose, mannose, fructose, rhamnose, glucosamine, xylose, arabinose, galactosamine, galacturonic acid	HPLC, HPIC	—
Zhou et al., 2024, (56) China	*L. plantarum* NMGL2	Pure	Unknown	Unknown	Unknown	Unknown
Niu et al., 2023, (43) China	*Bifidobacterium breve* H4-2	Crude	Not reported	Mannose, rhamnose, glucose, acetylglucosamine, galactose (1.00:0.76:1.79:1.03:0.63)	FT-IR	Total carbohydrates 80.73%; protein 4.31%; uronic acid 2.07%; sulfates 3.37%
Xie et al., 2023, (50) China	*Weissella cibaria* PFY06 isolated from strawberries	Pure	8.08 × 10^6^ Da	Glucose (primary component)	HPLC	Glucan linkage structure
Xie et al., 2023, (48) China	*M. purpureus* 40,269	Pure	Unknown	Unknown	Unknown	Unknown
Wang et al., 2022, (46) China	*L. plantarum* JLAU103	Unknown; received from the Laboratory of Fermentation Engineering, Jilin Agricultural University, Changchun, China	Unknown	Unknown	Unknown	Unknown
Wan et al., 2022, (45) China	*L. rhamnosus* ZFM231 from fresh milk	Crude	1.32 × 10^4^ Da	Mannose, galactose, glucosamine, glucose, glucuronic acid, rhamnose (7.08:2.61:2.44:2.22:1.59:1)	FTIR; NMR	Total sugar 44.00%; protein 3.65%; sulfate 12.16%; uronic acid 13.00%; primary residues 1,4-Manp, 1,2-Galp, 1,2-Glcp, 1,3,4-Glcp, 1,3-GlcNAc
Liu et al., 2021, (39) China	*L. helveticus* KLDS1.8701 isolated from fermented dairy products in Sinkiang, China	Pure	Unknown	Unknown	Unknown	Unknown
Ma et al., 2021, (41) China	*L. plantarum*-12	Crude	Unknown	Unknown	Unknown	Unknown
Noda et al., 2021, (29) Japan	*L. paracasei* IJH-SONE68	Pure	Unknown	Unknown	Unknown	Unknown
Kuang et al., 2020, (35) China	*Bacillus amyloliquefaciens* DMBA-K4 isolated from Yunnan Puer tea	Pure	10,067 Da	Mannose, rhamnose, glucuronic acid, glucose (40.09:23.65:11.42:17.68)	FT-IR	Second elution fraction used; described as relatively homogenous, acidic polysaccharide
Min et al., 2020, (42) China	*L. plantarum* YW11 isolated from Tibetan kefir	Pure	Unknown	Unknown	Unknown	Unknown
Ma et al., 2020, (40) China	*Lactobacillus fermentum* HNUB20 isolated from fermented seafood in Hainan area, China	Pure	45,065 Da	Not reported	Not reported	—
Chen et al., 2019, (33) China	*Streptococcus thermophilus* MN-BM-A01	Pure	4.23 × 10^5^ Da	Rhamnose, glucose, galactose, mannose (12.9:26.0:60.9:0.25)	Phenol-sulfuric acid assay	EPS-1 fraction used; described as a neutral polysaccharide
Zhou et al., 2019, (55) China	*L. plantarum* NCU116	Pure	3.84 × 10^5^ Da	Glucose, glucosamine, glucuronic acid, mannose, galactosamine (4:1.4:2:9.6:1)	Not reported (cited from prior work)	83.7% polysaccharide; 15.1% protein
Yu et al., 2018, (51) China	*Rhizopus nigricans* isolated from straw	Pure	Unknown	Unknown	Unknown	Unknown
Hamet et al., 2016, (31) Argentina	Kefiran isolated from CIDCA AGK1 kefir grains	Pure	Unknown	Unknown	Anthrone method; TLC; Bradford assay	Unknown
Li et al., 2014, (38) China	*Bifidobacterium bifidum* WBIN03	Unknown	Unknown	Unknown	Unknown	Unknown

“Unknown” indicates the attribute was not reported in the source manuscript. “Not reported” indicates the source manuscript characterized the EPS in some respect (e.g., molecular weight or purification method) but did not report that specific attribute. Disease model and corresponding intestinal health outcomes are reported by study in Table 3.

### 3.4 Intestinal health

#### 3.4.1 Intestinal epithelium and morphology

Histological analysis was used to assess intestinal tissue structure and evaluate the effects of EPS on tissue morphology (Table 3). In studies in which colonic tissue was challenged, EPS were shown to alleviate inflammation-induced shortening of the colon, at doses ranging from 10 to 600 mg/kg (29, 30, 33, 35, 39, 42, 45, 54–56). Similarly, EPS decreased the disease activity index (DAI) histopathologic scores reported in EPS doses from 10 to 600 mg/kg (29, 33, 35, 42, 45, 55) and restored crypt structure and depth in the colon visible in doses 50 through 400 mg/kg EPS (34, 47, 51). In cancer-induced models via azoxymethane and dextran sulfate sodium salt mixture, there was also reported to be a decrease in tumor number and size in EPS treatment groups compared to model control groups in doses of 150 and 200 mg/kg EPS (41, 51). The small intestine was also investigated for the morphometric effect of EPS on tissue structure. There was an increase (*p* < 0.05) in villus height and crypt depth in the small intestine with EPS doses 50, 100, and 200 mg/kg (46) and specifically the ileum in EPS dose 100 mg/kg (48). Goblet cells were a specific intestinal tissue attribute analyzed in the small intestines and colon as their key functionality is in protecting the intestinal epithelium, by producing and secreting mucin proteins (58). There was an increase (*p* < 0.05) in goblet cell count per cross section in the colon in EPS exposure doses ranging from 10 to 500 mg/kg (30, 35, 48, 51, 55, 56) and ileum at 100 mg/kg EPS dose (48).

**Table 3:** Characteristics of included manuscripts.

Author, Year, Country, Ref #	Animal model	Disease model	EPS Source and format	Groups and doses	Follow-up and dosing regimen	Primary outcomes	Secondary outcomes
Zhao et al., 2025, China (Ref 54)	SPF C57BL/6 J mice; male; 3 weeks old	High-fat diet (HFD)-induced obesity/insulin resistance	*Lactobacillus acidophilus* YL01; Pure	Control (normal diet); HFD (model, high-fat diet); HFD + YL01 (10^8^ CFU/mL); HFD + EPS-L (50 mg/kg); HFD + EPS-M (100 mg/kg); HFD + EPS-H (200 mg/kg)	12 weeks. Control: normal diet; all other groups fed HFD; YL01 group received 10^8^ CFU/mL via daily gavage; EPS groups received daily gavage of respective dose	Δ Simpson and Shannon diversity in gut microbiota; observed difference in Lactobacillus genus (not statistically significant); ↑ SCFA production	↓ body fat weight (epididymal, subcutaneous); ↓ histological liver lesions; ↓ blood glucose; ↓ IL-6 and TNFα in liver
Gao et al., 2025, China (Ref 34)	SPF C57BL/6 J mice; male; 8 weeks old	DSS-induced colitis	*Bacillus thuringiensis* IX-01; Pure	Control (normal diet + saline gavage); DSS (model, 2.5% DSS); BPS-2-L (50 mg/kg); BPS-2-M (100 mg/kg); BPS-2-H (200 mg/kg)	14 days, active intervention days 8–14. 2.5% DSS in drinking water days 8–14 (all but Control); daily oral gavage 200 μL saline or BPS-2 (50/100/200 mg/kg) same window	↑ ZO-1, OCLN, CLDN-1, IL-10, SOD, CAT, GSH-Px in colon; ↓ IL-1β, IL-6, TNFα, MPO, iNOS, COX-2 in colon; ↑ SCFA (acetate, propionate, butyrate); Δ crypt depth/goblet cell count improved; Δ PCoA/β-diversity improved	↑ IL-10, SOD, CAT, GSH-Px in serum; ↓ IL-1β, IL-6, TNFα, MPO, iNOS, COX-2 in serum
Wang et al., 2025, China (Ref 47)	C57BL/6 N mice; male; 4 weeks old	DSS-induced colitis	Levilactobacillus brevis M-10; Pure	Con (saline); Model/DSS (3.5% DSS); LD (EPS1 100 mg/kg); MD (EPS1 200 mg/kg); HD (EPS1 400 mg/kg); PC (5-ASA 100 mg/kg)	21 days. EPS groups daily gavage full 21 days; 3.5% DSS via drinking water Day 15–21 (all but Con); PC received saline first 14 days then 5-ASA concurrent with DSS Day 15–21	↑ CLDN-5, OCLN, IL-10 in colon; ↓ IL-6, IL-1β, TNFα, MPO in colon; Δ crypt structure/colon length; ↑ Shannon index; ↑ Firmicutes, ↓ Bacteroidota; ↑ SCFA (acetic, propionic, n-butyric acid)	↓ LPS and MPO in serum; ↔ WBC, RBC, HCT, MCV in serum; HD group restored normal body weight
Su et al., 2025, China (Ref 44)	C57BL/6 J mice; male; 6 weeks old	DSS-induced colitis	Lacticaseibacillus paracasei JY062; Crude	NC (normal control); DSS (model, 3% DSS); JY062 (10^8^ CFU *L. paracasei*); EPS (600 mg/kg); JEC (10^8^ CFU + EPS 600 mg/kg)	21 days. Intervention groups 200 μL daily gavage Day 8–14 prior to DSS; 3% DSS all but NC Day 15–21 (acute model); gavage continued daily throughout Day 15–21	↓ TLR4, MyD88, p65, p-p65, TNFα, IFNγ, IL-6, IL-17, IL-18 in colon; ↑ IκBα, IL-10, TGFβ in colon; ↑ SCFA (acetic, propionic, isobutyric, valeric acid)	↓ LPS, MPO in serum
Zhang et al., 2025, China (Ref 53)	SPF BALB/c mice; male; unknown age	Loperamide-induced constipation	Lacticaseibacillus rhamnosus LRa05; Crude (checked for quality)	NC (normal control); MC (model, loperamide); HK-L (postbiotic 6 × 10^8^ CFU/mL); HK-H (postbiotic 1.2 × 10^9^ CFU/mL); EPS-L (200 mg/kg); EPS-H (400 mg/kg)	14 days. Loperamide (10 mg/kg) daily Day 8–14 (all but NC) to induce constipation; 0.2 mL saline/postbiotic/EPS daily gavage 1 week; post-treatment split into black-stool and intestinal-propulsion test groups	↓ TNFα, IL-1β, IL-6 in colon; ↑ ZO-1, OCLN in colon; ↓ AChE, SS, AQP3, AQP4, AQP8 in colon; ↑ MTL, GAS, VIP, 5-HT in colon; ↑ Akkermansia and Muribaculaceae genera; ↓ Firmicutes; ↑ SCFAs (acetic, butyric, propionic acid)	↑ MTL, GAS, 5-HT, VIP in serum; ↓ SS, IL-6, IL-1β, TNFα in serum
Li et al., 2025, South Korea (Ref 30)	C57BL/6 mice; male; 6–8 weeks old	DSS-induced colitis	Lactiplantibacillus plantarum ZL1; Pure	Control; Model (3% DSS); EPS-W-1 Low (100 mg/kg), Medium (200 mg/kg), High (400 mg/kg); 5-ASA positive control (200 mg/kg)	13 days. 3% DSS drinking water first 3 days (all but Control); corresponding doses daily for 10 consecutive days following induction; 5-h fast Day 13 before euthanasia	↑ ZO-1, OCLN, IL-10 in colon; ↓ p-NF-κB, p-IκBα, TNFα, IL-1β, IL-6, MPO in colon; colon shortening alleviated; ↑ goblet cell count; ↑ Shannon and Chao1 indices; ↑ Akkermansia and Muribaculaceae genera; ↓ Escherichia-Shigella and Helicobacter; ↑ SCFA (acetic, propionic, butyric acid)	↓ LPS, TNFα, IL-1β, IL-6, MDA in serum; ↑ SOD in serum
Li, H, et al., 2024b, China (Ref 36)	SPF C57BL/6 J mice; male; 8 weeks old	DSS-induced colitis	*Bifidobacterium longum* YS108R / C11A10B; Crude	Control (saline); DSS (model, 2.5% DSS); Mesalazine (300 mg/kg); YS108R EPS (300 mg/kg); C11A10B EPS (300 mg/kg)	15 days. 2.5% DSS ad libitum Day 8–15 (all but Control); daily oral gavage (0.2 mL) Day 1–15; treatments given 7 days pre-DSS, continued 8 days during DSS	↑ ZO-1, OCLN, CLDN-1, MUC2, IL-10 in colon; ↓ p-p65, p-IκBα, TNFα, IL-1β, IL-6, MPO in colon; ↑ Bifidobacterium, Lactobacillus, Akkermansia genera; ↓ Escherichia-Shigella and Bacteroides; ↑ Shannon and Chao1 indices; ↑ SCFA (acetic, propionic, butyric acid)	↓ LPS, D-LA, TNFα, IL-1β, IL-6, MDA in serum; ↑ T-AOC, GSH-Px in serum
Xie et al., 2024, China (Ref 49)	SPF C57BL/6 mice; male; 6 weeks old	DSS-induced colitis	Monascus purpureus 40,269 (G-EMP); Pure	NC (normal control); MC (model, 3% DSS); 5-ASA (100 mg/kg); LG-EMP (50 mg/kg); HG-EMP (200 mg/kg)	14 days. 3% DSS drinking water final 7 days (all but NC); treatments daily oral gavage	↓ TLR4, p-p38, p-JNK, p-ERK, p-p65, p-IκBα, TNFα, IL-1β, IL-6, MPO in colon; ↑ ZO-1, OCLN, IL-10, MUC2 in colon; ↑ Shannon and Chao1 indices; ↑ Akkermansia and Muribaculaceae genera; ↓ Escherichia-Shigella, Bacteroides, Helicobacter; ↑ SCFA (acetic, propionic, butyric acid)	↓ LPS, D-LA, TNFα, IL-1β, IL-6, MDA in serum; ↑ IL-10, SOD, GSH-Px in serum
Li, H, et al., 2024a, China (Ref 37)	SPF C57BL/6 J mice; male; 8 weeks old	DSS-induced colitis	Limosilactobacillus mucosae CCFM1273; Crude	Control (saline); DSS (model, 2.5% DSS); CCFM1273 EPS (300 mg/kg)	15 days. CCFM1273 EPS group daily gavage (300 mg/kg) from Day 1, 7-day pretreatment before induction; 2.5% DSS Day 8–15 for DSS/EPS groups; treatments continued daily during 8-day DSS	↓ Fas, FasL, Caspase-3, TLR4, p-p65, p-IκBα, TNFα, IL-1β, IL-6, MPO in colon; ↑ ZO-1, OCLN, IL-10 in colon; ↑ Shannon and Chao1 indices; ↑ Akkermansia and Limosilactobacillus genera; ↓ Escherichia-Shigella and Bacteroides; ↑ SCFA (acetic, propionic, butyric acid)	↓ LPS, D-LA, MDA in serum; ↑ T-AOC and GSH-Px in serum
Chen et al., 2024, China (Ref 32)	Kunming mice; female; unknown age	Cyclophosphamide (CTX)-induced immunosuppression	Lacticaseibacillus rhamnosus ZFM216; Crude	NC (normal control); MC (model, CTX); PC (Lentinan); LD (100 mg/kg); MD (200 mg/kg); HD (300 mg/kg)	14 days. IP injection CTX (100 mg/kg BW) daily 3 consecutive days (all but NC); treatments via gavage daily 11 consecutive days	↑ Shannon and Chao1 indices; ↑ Lacticaseibacillus, Muribaculaceae, Lachnospiraceae_NK4A136_group genera; ↓ Staphylococcus and Helicobacter genera; ↑ SCFA (acetic, propionic, butyric acid)	↓ MDA in serum; restored spleen weight; ↑ IL-2, IL-6, TNFα, IFNγ, IgG, IgM, IgA, SOD, CAT, GSH-Px in serum
Zhang et al., 2024, China (Ref 52)	SPF C57BL/6 J mice; male; 6 weeks old	DSS-induced colitis	*L. helveticus* 6 M3/DQHXNQ5M22, *L. plantarum* VJLHD4L1/CCFM1393; Crude	Control (normal); DSS (model, 3% DSS); MnEPS-6 M3, MnEPS-Q5M22, MnEPS-D4L1, MnEPS-CCFM1393 (200 mg/kg/d each)	7 days. All groups (except Control) received 3% DSS and respective MnEPS treatment during the same 7-day period; 200 mg/kg/d daily gavage	↓ TLR4, p-p65/p65, MyD88, TNFα, IL-1β, IL-6, MPO in colon; ↑ ZO-1, OCLN, MUC2, IL-10 in colon; ↑ Shannon and Chao1 indices; ↑ Akkermansia, Muribaculaceae, Lachnospiraceae genera; ↓ Escherichia-Shigella and Helicobacter; ↑ SCFA (acetic, propionic, butyric acid)	↓ LPS, D-LA, IL-6, IL-1β, TNFα in serum
Zhou et al., 2024, China (Ref 56)	SPF BALB/c mice; male; 6 weeks old	DSS-induced colitis	*L. plantarum* NMGL2; Pure	NC (normal control); M (model, 5% DSS); P (sulfasalazine 0.45 g/kg); EPS-L (20 mg/kg); EPS-M (40 mg/kg); EPS-H (80 mg/kg)	21 days. 5% DSS drinking water 14 consecutive days (all but NC); treatments daily 7 days following induction, dosed by body weight	↑ ZO-1, OCLN, IL-10 in colon; ↓ p65, TNFα, IL-1β, IL-6, MPO in colon; restored colon length and goblet cell count; ↑ Shannon and Simpson indices; ↑ Lactobacillus, Akkermansia, Bacteroides genera; ↓ Escherichia-Shigella and Proteus genera	↑ IL-10 in serum; ↓ TNFα, IL-1β, IL-6 in serum; ↓ DAI score
Niu et al., 2023, China (Ref 43)	C57BL/6 J mice; male; 6 weeks old	DSS-induced colitis	*Bifidobacterium breve* H4-2; Crude	NC (normal control); DSS (model, 2.5% DSS); BBEL (200 mg/kg·d); BBEH (400 mg/kg·d)	14 days. All mice gavaged daily 0.2 mL (saline or BBE) full 14 days; Day 1–7 gavage with free water access; Day 8–14 continued gavage while DSS/BBE groups received 2.5% DSS in drinking water	↑ ZO-1, OCLN, IL-10 in colon; ↓ p-p65, TLR4, TNFα, IL-1β, IL-6, MPO in colon; ↑ Akkermansia, Muribaculaceae, Lactobacillus genera; ↓ Escherichia-Shigella and Helicobacter; ↑ SCFA (acetic, propionic, butyric acid)	↓ TNFα, IL-1β, IL-6, MDA in serum; ↑ SOD, GSH-Px, T-AOC in serum
Y. Xie et al., 2023, China (Ref 50)	C57BL/6 J mice; male; 6–8 weeks old; n = 10	High-fat diet (HFD)-induced obesity	*Weissella cibaria* PFY06; Pure	ND (normal diet control); HFD (high-fat diet model); PC (Orlistat 15.6 mg/kg/d); WCL (PFY06-EPS low, 100 mg/kg/d); WCH (PFY06-EPS high, 400 mg/kg/d)	8 weeks (after 2-week HFD screening). 60 mice fed 60 kcal% HFD 2 weeks; bottom 1/3 (obesity-resistant) eliminated, leaving 40 obesity-sensitive mice; 4 HFD groups + ND followed 8 further weeks; treatments daily by body weight	↓ Firmicutes/Bacteroides ratio; ↑ Bacteroides, Prevotella, Roseburia, Oscillibacter genera; ↑ SCFA (acetic, propionic, butyric acid); ↓ hydrogen sulfide; restored epithelial integrity	↓ fasting blood glucose, insulin resistance index, leptin, TNFα, IL-6, LPS in serum; ↑ insulin, insulin sensitivity index, HDL-C, GLP-1, PYY, CCK in serum
L. Xie et al., 2023, China (Ref 48)	SPF C57BL/6 J mice; male; 6 weeks old	Cyclophosphamide (Cy)-induced intestinal injury	Monascus purpureus 40,269 (Cy model); Pure	NC (normal control); MC (model, Cy); G-EMP (Cy + G-EMP, 100 mg/kg)	19 days. 80 mg/kg BW cyclophosphamide IP daily 5 consecutive days (all but NC); following induction, 100 mg/kg G-EMP (or saline) daily gavage 14 days	↑ goblet cell count in ileum/colon; restored villus length and crypt depth in ileum; ↑ ZO-1, IL-10, OCLN, MUC2 in colon; ↓ PI3K, AKT, MAPKs, NF-κB, TNFα, IL-6, IL-1β in colon; ↑ SCFA (acetic, propionic, butyric acid); ↑ Shannon and Chao1 indices; ↑ Bifidobacterium, Lactobacillus, Akkermansia genera; ↓ Staphylococcus and Helicobacter genera	↓ MDA, ↑ GSH-Px/T-AOC in liver and serum; ↓ TNFα, IL-6, IL-10, IL-1β in serum; normalized spleen index
Wang et al., 2022, China (Ref 46)	SPF BALB/c mice; female; 6–8 weeks old	Cyclophosphamide (Cy)-induced immunosuppression	Lactiplantibacillus plantarum JLAU103; Unknown	NC (normal control, saline); MC (model, Cy); LD (EPS103 50 mg/kg); MD (100 mg/kg); HD (200 mg/kg); PC (Levamisole HCl 40 mg/kg)	12 days. 80 mg/kg BW Cy IP daily 3 consecutive days (all but NC); treatments via gavage once daily 9 days	↑ villus height and crypt depth in small intestine; ↑ ZO-1, OCLN, CLDN-1, MUC2, Nrf2, p-p38, p-JNK, p-ERK, sIgA, CAT, SOD, GSH-Px in small intestine; ↓ Keap1, MDA in small intestine; ↑ Lactobacillus, Akkermansia, Muribaculaceae genera; ↓ Staphylococcus and Helicobacter; ↑ SCFA (acetic, propionic, butyric acid)	restored spleen weight; ↓ MDA in serum; ↑ T-AOC, IgG, IgM, CAT, SOD, GSH-Px, IL-2, IL-4, IL-6, IFNγ, TNFα in serum
Wan et al., 2022, China (Ref 45)	C57BL/6 mice; female; unknown age	DSS-induced colitis	*Lactobacillus rhamnosus* ZFM231; Crude	A (control, normal); B (DSS model, 2.5% DSS); C (mesalazine 200 mg/kg); D (EPS low, 100 mg/kg); E (EPS medium, 300 mg/kg); F (EPS high, 600 mg/kg)	14 days. 2.5% DSS ad libitum first 7 days (Groups B–F); following induction, treated an additional 7 days (Day 8–14)	↑ colon length via alleviating shortening; ↓ histopathology score in colon; ↑ SCFA (acetic, propionic, butyric acid); ↑ Shannon and Chao1 indices; ↑ Bacteroidetes phyla; ↓ Firmicutes and Proteobacteria phyla; ↑ Muribaculaceae_unclassified, Bacteroides, Alloprevotella genera; ↓ Escherichia-Shigella and Turicibacter genera	↓ DAI score; ↑ body weight
Liu et al., 2021, China (Ref 34)	C57BL/6 mice; male; 7 weeks old	DSS-induced colitis	*Lactobacillus helveticus* KLDS1.8701; Pure	NC (normal control, PBS); DSS (model, 3.0% DSS); EPS-1 (200 mg/kg)	21 days. EPS-1 group daily oral gavage (200 mg/kg) 14 days prior to induction; 3.0% DSS drinking water Day 15–21 for DSS/EPS-1 groups; NC and DSS received equal volumes sterile PBS via gavage daily	↑ ZO-1, CLDN-1, OCLN, MUC2, IL-10 in colon; ↓ TLR4, p-p65, p-p38, p-JNK, p-ERK, TNFα, IL-1β, IL-6, MPO in colon; ↑ colon length via alleviating shortening; ↑ Shannon and Chao1 indices; ↑ Akkermansia, Lactobacillus, Muribaculaceae genera; ↓ Escherichia-Shigella, Bacteroides, Turicibacter; ↑ SCFA (acetic, propionic, butyric acid)	↓ TNFα, IL-1β, IL-6, LPS in serum; ↑ IL-10 in serum
Ma et al., 2021, China (Ref 36)	C57BL/6 mice; male; 6 weeks old	AOM/DSS-induced colitis-associated colon cancer	Lactiplantibacillus plantarum-12; Crude	N_Con (normal control, saline); M_Con (model, AOM/DSS + saline); M_5ASA (5-ASA 75 mg/kg); M_EPS (LPEPS, 200 mg/kg)	12 weeks. Single IP injection AOM (12.5 mg/kg) Day 0, followed by 3 cycles of 2.5% DSS drinking water (5 days each) weeks 2, 6, 9; daily oral gavage throughout 12-week period	↓ tumor number and size in colon; ↑ ZO-1, OCLN, CLDN-1, MUC2, IL-10 in colon; ↓ p-p65, p-p38, p-JNK, p-ERK, STAT3, TNFα, IL-1β, IL-6 in colon; ↑ Akkermansia, Muribaculaceae, Lactobacillus genera; ↓ Escherichia-Shigella, Desulfovibrio, Staphylococcus genera; ↑ SCFA (acetic, propionic, butyric acid)	normalized spleen index; ↓ LPS, IL-6, TNFα in serum; ↑ IL-10 in serum
Noda et al., 2021, Japan (Ref 24)	SPF C57BL/6 J Jms Slc mice; male; 7 weeks old	DSS-induced ulcerative colitis	*Lactobacillus paracasei* IJH-SONE68; Pure	NC (negative control, no UC induction); PC (positive control, DSS only); N-EPS (DSS + Neutral-EPS); A-EPS (DSS + Acidic-EPS)	21 days. EPS solutions (50 mg/kg) administered 7 days prior to UC induction; 3% DSS added to drinking water for all but NC, 14 days	↓ histopathology score in colon; ↑ colon length via alleviating shortening; ↓ TNFα, IL-1β, IL-6, MPO, p-IκBα, IκBα, IL-12p40 in colon; ↑ IL-10 in colon	↓ DAI score; ↑ body weight; ↓ TNFα, IL-6 in serum
Kuang et al., 2020, China (Ref 30)	SPF BALB/c mice; male; 7–8 weeks old	DSS-induced colitis	*Bacillus amyloliquefaciens* DMBA-K4; Pure	Control (distilled water gavage); DSS (4% DSS + distilled water gavage); DSS + EPS Low (80 mg/kg EPS-K4); DSS + EPS High (160 mg/kg EPS-K4)	7 days. 4% (w/v) DSS drinking water 7 consecutive days (all but Control); EPS-K4 or distilled water via daily intragastric gavage concurrent with DSS induction (Day 1–7)	↑ colon length via alleviating shortening and increased goblet cell count; ↓ histopathology score in colon; ↑ MUC2 and IL-10 in colon; ↓ TNFα, IL-1β, IL-6, MPO, iNOS, COX-2 in colon; ↑ Shannon and Chao1 indices; ↑ Lactobacillus and Akkermansia genera; ↓ Escherichia-Shigella and Enterococcus genera	↓ TNFα, IL-1β, IL-6 in serum; ↑ IL-10 in serum; ↓ DAI score; ↑ body weight
Min et al., 2020, China (Ref 37)	ICR mice; male; unknown age	DSS-induced colitis	*Lactobacillus plantarum* YW11; Pure	B (blank, healthy control); N (5% DSS + saline 20 mL/kg); P (5% DSS + prednisolone 5 mg/kg); YW11-L (10 mg/kg); YW11-M (25 mg/kg); YW11-H (50 mg/kg)	21 days. 5% DSS administered Day 1–14 to establish bowel inflammation; treatments once daily Day 15–21	↑ IL-10 and TGFβ in colon; ↓ TNFα, IL-1β, IL-6, IFNγ, IL-12, IL-18 in colon; ↓ histopathology score in colon; ↑ colon length via alleviating shortening; ↑ Roseburia, Ruminococcus, Blautia genera	↓ DAI score; ↑ body weight
Ma et al., 2020, China (Ref 35)	Zebrafish; male and female; adult (3 months); 16 fish/tank, 3 tanks/treatment	None (healthy zebrafish; feed supplementation)	*Lactobacillus fermentum* HNUB20; Pure	Control (standard commercial feed); EPS (control diet + 1% m/m crude EPS); HNUB20 (control diet + *L. fermentum* HNUB20, 8.0 log₁₀ CFU/g)	21 days. EPS or probiotic cells mixed directly into commercial feed; daily feeding at 3% of total body weight; water replaced daily 9 a.m.; whole intestines used (esophagus to anus)	↓ TNFα, IL-1β in intestines; ↑ IL-10, ZO-1 in intestines; ↑ SCFAs (acetate, propionate, butyrate); ↑ Pelomonas (EPS group) and Lactobacillus (HNUB20 group); ↓ Ochrobactrum, Sediminibacterium, Sphingomonas	Not evaluated
Chen et al., 2019, China (Ref 28)	SPF BALB/c mice; male; 6–8 weeks old	DSS-induced colitis	*Streptococcus thermophilus* MN-BM-A01; Pure	Control (distilled water gavage); DSS Alone (2.5% DSS Day 1–7 + distilled water Day 8–14); EPS-1 Low (2.5% DSS + 20 mg/kg EPS Day 8–14); EPS-1 High (2.5% DSS + 200 mg/kg EPS Day 8–14)	14 days. 2.5% DSS drinking water first 7 days to establish colitis; EPS-1 (20 or 200 mg/kg) daily oral gavage only during second 7 days (Day 8–14)	↓ histopathology score in colon; ↑ colon length via alleviating shortening; ↑ ZO-1, OCLN, IL-10 in colon	↓ DAI score; ↑ body weight
Zhou et al., 2019, China (Ref 50)	C57BL/6 mice; male; 8 weeks old	DSS-induced colitis	*Lactobacillus plantarum* NCU116; Pure	Control (standard chow + normal water); DSS (4% DSS + 0 mg/kg EPS, vehicle only); EPS116 Low (80 mg/kg); EPS116 High (160 mg/kg)	7 days. 4% DSS drinking water for all but healthy control; EPS116 dissolved in 100 μL PBS, daily oral gavage	↑ colon length via alleviating shortening and goblet cell count; ↓ histopathology score in colon; ↑ ZO-1, OCLN, MUC2, Math1, Klf4, c-Jun/p-c-Jun, Villin in colon; ↓ Hes1, MPO in colon	↓ DAI score; ↑ body weight
Yu et al., 2018, China (Ref 46)	BALB/c mice; unknown sex; 4–6 weeks old	AOM/DSS-induced colitis-associated colon cancer	Rhizopus nigricans; Pure	Normal control (regular food/water); AOM/DSS Only (model, normal saline); AOM/DSS + EPS1-1 (150 mg/kg)	12 weeks. Single IP injection AOM (10 mg/kg) Day 1; three cycles of 2% DSS drinking water (7 days) followed by 14 days regular water recovery; EPS1-1 daily oral gavage from Day 1 through 14 days after model established	↓ tumor size and number in colon; ↓ incidence of neoplasia; ↑ villus height, goblet cell count, crypt depth in colon; ↑ MUC2 in colon; ↑ SCFAs (butyric acid); ↑ Bacteroides, Akkermansia, Lactobacillus genera; ↓ Desulfovibrio and Helicobacter genera	↑ body weight
Hamet et al., 2016, Argentina (Ref 26)	BALB/c mice; female; 6–8 weeks old	None (healthy mice; microbiota modulation)	Kefiran / CIDCA AGK1 kefir grains; Pure	Control (conventional diet + sterile drinking water); Kefiran Treatment (conventional diet + kefiran solution, 300 mg/L in drinking water)	21 days. Fresh kefiran solutions prepared daily by dissolving lyophilized kefiran in sterile water; 300 mg/L administered ad libitum (~0.75–1 mg kefiran/animal/day); feces collected Days 0, 2, 7, 14, 21	↑ Bifidobacteria and Lactobacilli genera; ↔ total Eubacteria domain; ↑ SCFAs (acetic, propionic, lactic acid); post-kefiran exposure returned microbiota to baseline	Not evaluated
Li et al., 2014, China (Ref 33)	BALB/c mice; female; 6–8 weeks old	None (healthy mice; microbiota modulation)	*Bifidobacterium bifidum* WBIN03; Unknown	Control (sterile water); Low-EPS (50 mg/kg/d); High-EPS (500 mg/kg/d)	28 days. EPS diluted with sterile deionized water to 10 and 100 mg/mL concentrations; daily oral intervention over 4 weeks	↑ Shannon index for microbial diversity; ↑ Lactobacilli genera; ↓ Enterobacteria genera	↔ body weight; ↑ Lactobacilli and Bifidobacteria live cell counts in stool; ↓ Enterobacteria and *Bacteroides fragilis* live cell counts in stool

EPS source and crude/purified status cross-referenced from Table 2. Reference numbers correspond to the manuscript reference list.

#### 3.4.2 Intestinal tight junctions and barrier integrity

Tight junction proteins (TJPs) are responsible for maintaining intestinal epithelial integrity by regulating the passage of molecules across the epithelium into the bloodstream (59). Zona occluden (ZO-1) is an intracellular scaffolding protein responsible for anchoring tight junctions to the cytoskeleton (59). Small intestinal ZO-1 expression was significantly increased (*p* < 0.05) in EPS treatment groups compared with controls (46), and generally in the gastrointestinal tract (GIT), shown only in the zebrafish model (40), and in the colon (30, 33, 34, 37, 39, 41, 43, 49, 50, 52, 53, 55, 56). Occludin’s (OCLN) function is to regulate paracellular transport between adjacent cells and maintain the structural integrity of the intestinal epithelium (59). Colon OCLN expression was significantly increased (*p* < 0.05) in EPS treatment groups compared with controls (30, 33, 34, 36, 37, 39, 41, 43, 47–49, 52, 53, 55, 56) and small intestine (46), relative to model control groups. There are several types of claudin proteins (CLDN) that maintain the intestinal epithelium through sealing intercellular space (59, 60) in the intestinal epithelium with an increase (*p* < 0.05) in expression of CLDN-5 in the colon (47), CLDN-1 in the colon (34, 36, 39, 41), and CLDN-1 in the small intestine (46). Additionally, other proteins such as mucins (MUC) act as a physical barrier to protect the brush border membrane. MUC2 specifically secretes mucus that protects the microvilli which increased (*p* < 0.05) in the colon (35, 36, 39, 41, 48, 49, 51, 52, 55) and the small intestine at EPS doses of 50, 100, and 200 mg/kg (46).

#### 3.4.3 Intestinal inflammatory cytokines

The inflammatory cytokines most frequently evaluated across the studies included in this review were tumor necrosis factor-alpha (TNF-α), interleukin (IL), and interferon-gamma (IFN-γ), which play key roles in immune signaling in both pro- and anti-inflammatory pathways (61) (Table 3). Overall, all reviewed manuscripts agree upon a decrease in TNF-α in the colon (seen in a range of EPS from 10 mg/kg to 600 mg/kg doses) (29, 30, 34–37, 39, 41–44, 47–49, 52, 53, 56) and a decrease in TNF-α in the GIT from zebrafish (EPS added in 1% m/m in feed) (40). Additionally, the manuscripts noted a decrease in IL-6 in the colon seen in EPS doses ranging from 10 mg/kg to 600 mg/kg (29, 30, 34–37, 39, 41–44, 47–49, 52, 53, 56), a decrease in IL-1β in the colon regardless of EPS dose (ranging from 10 to 400 mg/kg EPS) (29, 30, 34–37, 39, 41–43, 47–49, 52, 53, 56) and the general GIT intestines sample from the zebrafish model (1% EPS m/m in feed) (40). IL-10 was another inflammatory cytokine widely investigated by the manuscripts in this review, with the manuscripts resulting in an increase (*p* < 0.05) in IL-10 in the colon evaluated at a large range of EPS doses (10 to 600 mg/kg) (29, 30, 33–37, 39, 41–44, 47–49, 52, 56) and general GIT tissue samples (from the zebrafish model, 1% m/m of EPS in feed) (40). Other inflammatory cytokines and results reported in the reviewed manuscripts were a decrease (*p* < 0.05) in IFN-γ (42, 44) at 10, 25 and 50 mg/kg EPS and 600 mg/kg EPS respectively, a decrease (*p* < 0.05) in IL-12 at 10, 25 and 50 mg/kg EPS doses (29, 42), a decrease (*p* < 0.05) in IL-17 (600 mg/kg EPS) (44), and a decrease (*p* < 0.05) in IL-18 (42, 44). Conversely, TGFβ expression did increase (*p* < 0.05) in the colon at 600 mg/kg EPS dose (44).

#### 3.4.4 Intestinal signaling pathways

Several signaling pathways were investigated across the included studies, including the TLR4-MyD88 signaling pathway, NF-κB pathway, mitogen-activated protein kinase (MAPK) and Keap1-Nrf2 pathway, and the PI3K/AKT pathway (Table 3). EPS treatment was associated with downregulation of TLR4 expression (ranging from 50 to 600 mg/kg EPS doses) (37, 39, 43, 44, 49, 52) and MyD88 (at 200 and 600 mg/kg EPS dose) (44, 52) in the colon. The NF-κB pathway was seen to have a decrease (*p* < 0.05) in NF-κB expression in the colon at EPS administered in doses 100, 200 and 500 mg/kg (30, 48), a decrease in p65/p-p65 in the colon at EPS doses ranging from 50 to 500 mg/kg (37, 39, 41, 43, 44, 49, 52, 56), and a general trend in decreasing IκBα/p-IκBα in the colon at EPS doses ranging from 50 to 500 mg/kg (29, 30, 37, 49) but an increase (*p* < 0.05) in IκBα/p-IκBα in the colon was reported in one study where 600 mg/kg dose of EPS was evaluated (44). Additionally, the MAPK pathway saw a downregulation with EPS treatment in the colon via the p−/p38 gene (39, 41, 48, 49), the p-/JNK gene (39, 41, 48, 49), and p-/ERK gene (39, 41, 48, 49) in EPS doses ranging from 50 to 200 mg/kg. However, in small intestine tissue samples, there was an increase (*p* < 0.05) in p−/p38, p-/JNK, and p-/ERK genes with EPS treatment at all evaluated doses, 50, 100, and 200 mg/kg (46). In the same study, there was modulation of the Nrf2/Keap1 pathway in the small intestine with an increase (*p* < 0.05) in Nrf2 (46) and a decrease in Keap1 (46) at all three evaluated doses (50, 100, 200 mg/kg). Similarly, the PI3K/AKT pathway has inverse effects with an increase (*p* < 0.05) in PI3K expression (48) and a decrease in AKT expression (48) due to EPS treatment (100 mg/kg) compared to the model control in the colon.

#### 3.4.5 Intestinal oxidative stress, enzymes, and other intestinal genes

EPS were shown to decrease myeloperoxidase (MPO) levels in the colon across all of the studies that evaluated this oxidative stress marker, at doses ranging from 50 to 500 mg/kg (29, 30, 34–37, 39, 43, 47, 49, 52, 55, 56). There was an increase (*p* < 0.05) in antioxidant enzymes SOD, CAT, GSH-Px in both the small intestine with peak antioxidant enzyme activity at 200 mg/kg dose of EPS for all three genes (46) and colon seen in 50, 100, and 200 mg/kg EPS treatments (34).

Other gene expression changes associated with EPS treatment included an increase (*p* < 0.05) in transcription and differentiation-related genes in the colon. EPS treatment also led to a decrease in water channels proteins AQP4, AQP8 in the colon compared with DSS model controls across all tested doses (50, 100, and 200 mg/kg) (53). In addition, an increase (*p* < 0.05) in gut hormones, including MTL, GAS, VIP, 5-HT, was observed in the colon regardless of EPS dose (50, 100, and 200 mg/kg) (53). A decrease in apoptosis-related genes such as Fas, FasL, and Caspase-3 was observed in the colon (36). Finally, an increase (*p* < 0.05) in p−/c-Jun expression in colon was reported at 200 mg/mL EPS relative to model control (55).

#### 3.4.6 Intestinal microbiota

Microbiome data were reported in several ways across included manuscripts and are summarized in Table 3. Commonly used approaches included alpha and beta diversity analyses and taxonomic profiling at the phylum, family, and genus levels. A shift in viable cell counts in stool samples was reported in only one study (*n* = 1), which observed decreases in *Enterobacteria* and *Bacteroides fragilis* (38). Alpha and beta diversity metrics are used to characterize changes in microbial community composition and diversity (62). Alpha diversity is commonly assessed using indices such as the Shannon index, Chao1 index, and the Simpson index, which quantify community richness and evenness, species richness, and species dominance, respectively (62–64). EPS treatment was shown to significantly increase (*p* < 0.05) the Shannon index (30, 32, 35–39, 45, 47–49, 52, 54, 56), Chao1 index (30, 32, 35–37, 39, 45, 48, 49, 52), and Simpson index (54, 56) which are all measures of alpha diversity. Additionally, a significant shift in beta diversity was observed in principal coordinates analysis (PCoA) with tighter clustering within EPS treatment groups (34), suggesting a more uniform microbial community structure.

Phylum level taxonomic shifts in the microbiota across the included manuscripts showed mixed effects on the Firmicutes to Bacteroidetes (F/B) ratio. Reported changes included an increase (*p* < 0.05) in Bacteroidetes with a decrease in Firmicutes (45), an increase (*p* < 0.05) in Firmicutes with a decrease in Bacteroidetes (47), a decrease in Firmicutes alone (53), and an overall decrease in the F/B ratio (50). In addition, a reduction in relative abundance of Proteobacteria was reported in EPS treatment groups (45).

At the family level, an increase (*p* < 0.05) in the *Lactobacillaceae* family was reported (31, 35, 38–41, 43, 46, 48, 51, 56) and an increase in the *Muribaculaceae* family (*p* < 0.05) (30, 32, 39, 41, 43, 45, 46, 49, 52, 53).

At the genus level, an increase (*p* < 0.05) in beneficial genera, such as *Bacteroides*, was reported (45, 50, 51, 56), an increase (*p* < 0.05) in *Bifidobacterium* (31, 36, 38, 50), an increase (*p* < 0.05) in *Akkermansia* (30, 35–37, 39, 41, 43, 46, 49–53, 56), and an increase (*p* < 0.05) in SCFA producers such as *Roseburia*, *Ruminococcus*, and *Blautia* (32, 42, 48, 52). In contrast, pathogenic or inflammatory genera, such as *Escherichia,* were reduced in EPS treatment groups (30, 35–37, 39, 41, 43, 45, 49, 52, 56), *Helicobacter* (30, 32, 43, 46, 48, 49, 51, 52), *Staphylococcus* (32, 41, 46, 48), *Desulfovibrio* (41, 51), *Enterococcus* (35, 38), and *Bacteroides* (36, 37, 39, 49).

#### 3.4.7 Compositional analyses of short-chain fatty acids in fecal samples

The SCFA profile was evaluated based on changes in concentration and composition in the colon following EPS exposure (Table 3). The results of the manuscripts included in this systematic review demonstrate that EPS are associated with an overall increase (*p* < 0.05) in SCFAs (30–32, 36, 37, 39, 40, 43–54). Compositional shifts were associated with an increase (*p* < 0.05) in acetic acid (30–32, 36, 37, 39, 40, 43–50, 52, 53), an increase (*p* < 0.05) in propionic acid (30–32, 36, 37, 39, 40, 43–50, 52, 53), an increase (*p* < 0.05) in butyric acid (30, 32, 36, 37, 39, 40, 43, 45–53), an increase (*p* < 0.05) in lactic acid (31), an increase (*p* < 0.05) in valeric acid (44), and a decrease (*p* < 0.05) in hydrogen sulfide (50).

### 3.5 Secondary outcomes

Secondary health outcomes associated with EPS in the included manuscripts focused on immunomodulatory effects in the liver and spleen, as well as systemic health markers such as body weight and histopathology (Table 3). These outcomes were investigated using various approaches in 26 of the 28 manuscripts included in this review (29, 30, 32–39, 41–56).

#### 3.5.1 Body weight and comprehensive disease activity index

Body weight was evaluated in 10 of the manuscripts included in this review. The majority (8 of 10) were conducted in DSS-induced colitis mouse models, in which body weight significantly increased (*p* < 0.05), reflecting on restoration toward normal levels (29, 33, 35, 42, 45, 47, 51, 55). One study evaluated body weight in terms of adiposity in a high-fat diet-induced obesity mouse model (54), reporting decreases in both epididymal and subcutaneous fat mass. Conversely, in a healthy (unchallenged) model, no adverse effects on body weight were observed (38).

Comprehensive DAI scores were used to summarize the effects of EPS in DSS-induced colitis mouse models. EPS treatment was associated with a decrease in DAI scores (29, 33, 35, 39, 42, 45, 55, 56).

#### 3.5.2 Liver functional analyses

Two manuscripts (48, 54) investigated hepatic outcomes in different models, including a high-fat diet-induced obesity and insulin resistance model (54) and a cyclophosphamide-induced (Cy) intestinal injury model (48). In the high-fat diet model (54), the liver index was slightly elevated; however, EPS treatment at low, medium, and high doses restored the index towards normal levels (54). Histological analysis of the liver indicated that EPS alleviated tissue lesions, reduced lipid droplets accumulation, and helped maintain normal hepatocellular morphology (54). Malondialdehyde (MDA) was assessed as a biomarker of hepatic oxidative stress. EPS treatment significantly reduced (*p* < 0.05) MDA levels in the liver compared with the high-fat diet control group (54). Free fatty acids (FFA) were assessed in the liver in the context of a high-fat diet, and EPS treatment significantly reduced (*p* < 0.05) hepatic FFA levels (54). EPS were also investigated for their anti-inflammatory effects in the liver, with IL-6 and TNF-α significantly reduced (*p* < 0.05) compared with the high-fat diet control group (54). Lastly, the AMPK/ACC pathway was investigated in relation to gut microbiome biomarkers and SCFA metabolism, as well as hepatic AMPK/ACC pathway activation. Gene expression analysis in the liver showed significant activation (*p* < 0.05) of this pathway at the highest EPS dose.

In the Cy-induced intestinal injury model, the liver disease activity index was significantly reduced (*p* < 0.05) in the EPS treatment group compared with the model control group, indicating a restoration toward healthy baseline levels (48). Qualitative histological analysis of the liver also indicated normal hepatic lobule structure compared with the model control group (48). Additionally, EPS treatment significantly increased hepatic antioxidant activity compared with the model control group (*p* < 0.05), as evidenced by elevated superoxide dismutase (SOD), total antioxidant capacity (T-AOC), and catalase (CAT) levels (48). Conversely, MDA levels were significantly reduced (*p* < 0.05) in the EPS treatment group compared with the model control group, indicating reduced cellular damage (48).

#### 3.5.3 Spleen histomorphology

The spleen was assessed in several manuscripts to evaluate immune-related effects of EPS treatment under intestinal disease challenged conditions. Histological analysis indicated that EPS treatment normalized the spleen disease activity index (41, 48), and restored spleen weight (32, 46).

#### 3.5.4 Inflammatory and oxidative stress serum biomarkers

Inflammatory markers were assessed in serum to evaluate the effects of EPS on systemic inflammation. Most manuscripts reported a decrease in TNF-α levels following EPS treatment (29, 30, 34–36, 39, 41, 43, 48–50, 52, 53, 56). However, two manuscripts reported a significant increase (*p* < 0.05) in TNF-α relative expression (32, 46). Similarly, IL-6 exhibited the same trend, with 14 manuscripts reporting a decrease in IL-6 levels (29, 30, 34–36, 39, 41, 43, 48–50, 52, 53, 56). In addition, the same 2 manuscripts reported a significant increase (*p* < 0.05) in IL-6 levels (32, 46). Another gene showing conflicting expression patterns was IL-10 with several studies reporting an increase (*p* < 0.05) (34, 35, 39, 41, 49, 56) and others reporting a decrease (*p* < 0.05) (48). Other inflammation-related biomarkers modulated by EPS treatment included increase (*p* < 0.05) levels of IFNγ (32, 46), in IL-2 (32, 46), and IL-4 (46).

Oxidative stress and enzymatic activity were also evaluated with serum samples. Across the studies included in this review, a consistent trend of a decrease in MDA levels (*p* < 0.05) (30, 32, 36, 37, 43, 46, 49, 50), an increase in SOD (30, 32, 34, 43, 46, 49), an increase in serum glutathione peroxidase (GSH-Px) (*p* < 0.05) (34, 36, 37, 43, 46, 49), an increase in T-AOC (*p* < 0.05) (36, 37, 43, 46, 48), an increase in CAT (*p* < 0.05) (32, 34, 46), a decrease in myeloperoxidase (MPO) (*p* < 0.05) (34, 44), a decrease in inducible nitric oxide synthase (iNOS) (*p* < 0.05) (34), and a decrease in cyclooxygenase-2 (COX-2) (*p* < 0.05) (34).

Intestinal barrier function was evaluated using serum biomarkers, including lipopolysaccharides (LPS) levels (30, 34, 36, 37, 39, 41, 44, 47, 49, 50, 52) and D-lactate (D-LA) (36, 37, 49, 52) which both decreased (*p* < 0.05) in EPS treatment groups compared with model control groups.

Metabolic biomarkers were investigated in two manuscripts, focusing on pathways related to satiety, and obesity (50, 53). Motilin (MTL) and vasoactive intestinal peptide (VIP), gastrointestinal peptides involved in motility and function, were increased in EPS treatment groups (53). In the same study, serotonin (5-HT) was also evaluated as a marker of intestinal health modulation and was significantly increased (*p* < 0.05) in EPS treatment groups compared with model controls (53). Satiation hormones, including glucagon-like peptide-1 (GLP-1), peptide YY (PYY), and cholecystokinin (CCK), were significantly increased (*p* < 0.05) in EPS treatment groups in a high-fat diet model (50). In high-fat diet-induced obesity model, EPS treatment significantly reduced (*p* < 0.05) blood glucose levels (50, 54), decreased (*p* < 0.05) insulin resistance and leptin levels, and increased (*p* < 0.05) insulin and high density lipoprotein cholesterol levels (50).

Other serum immunological biomarkers included were increased (*p* < 0.05) levels of IgG and IgM (32, 46), as well as IgA (32). Additionally, EPS treatment normalized white blood cell count, red blood cell count, hematocrit, and mean corpuscular volume (34). Lastly, alanine aminotransferase (ALT) and aspartate aminotransferase (AST) levels were significantly elevated (*p* < 0.05) in mice fed a high-fat diet (HFD), whereas EPS treatment at all doses significantly reduced (*p* < 0.05) these levels, indicating a reduced liver injury (54).

### 3.6 Risk of bias

The results generated from the RoB analysis can be seen in Table 4. The domains of allocation concealment, random housing, and blinding of performance bias were unclear for all manuscripts, resulting in unknown bias. Baseline characteristics and incomplete outcome bias were low bias (100%) with no noted missing data or animals unaccounted for in the analysis. One study (3.57%) was marked as unclear for other sources of bias due to not enough information regarding analytical techniques to know the bias. Random outcome assessment was ranked as low bias for 2 of the 28 manuscripts (7.14%) primarily due to explicit language acknowledging complete cohort and/or all animals were evaluated rhetoric. Similarly, 2 of 28 manuscripts identified in this review were marked as low risk of bias for blinding (detection bias) due to the acknowledgment of blind histological analysis and/or multiple personnel. Lastly, 13 manuscripts (of 28) were marked as low risk in sequence generation and the remaining 15 (53.6%) were unclear.

**Table 4:** Summary of authors judgement for each risk bias following SYRCLE (Systematic Review Centre for Laboratory Animal Experimentation).

Author, Year (Ref #)	Sequence generation	Baseline characteristics	Allocation concealment	Random housing	Blinging (performance bias)	Random outcome assessment	Blinding (detection bias)	Incomplete outcome data	Selective outcome reporting	Other sources of bias
Zhao, et al., 2025 (54)	?	+	?	?	?	?	?	+	+	+
Gao, et al., 2025 (34)	+	+	?	?	?	?	?	+	+	+
Wang, et al., 2025 (47)	+	+	?	?	?	?	?	+	+	+
Su, et al., 2025 (44)	+	+	?	?	?	?	?	+	+	+
Zhang, et al., 2025 (53)	+	+	?	?	?	?	?	+	+	+
Li, et al., 2025 (30)	?	+	?	?	?	?	?	+	+	+
Li, et al., 2024a (36)	+	+	?	?	?	?	?	+	+	+
Xie, et al., 2024 (49)	+	+	?	?	?	+	+	+	+	+
Li, et al., 2024b (37)	+	+	?	?	?	?	?	+	+	+
Chen, et al., 2024 (32)	+	+	?	?	?	?	?	+	+	+
Zhang, et al., 2024 (52)	+	+	?	?	?	?	?	+	+	+
Zhou, et al., 2024 (56)	?	+	?	?	?	?	?	+	+	+
Niu, et al., 2023 (43)	+	+	?	?	?	?	?	+	+	+
Xie, Y, et al., 2023 (50)	?	+	?	?	?	?	?	+	+	+
Xie, L, et al., 2023 (48)	+	+	?	?	?	?	?	+	+	+
Wang, et al., 2022 (46)	?	+	?	?	?	?	?	+	+	+
Wan, et al., 2022 (45)	?	+	?	?	?	?	?	+	+	+
Liu, et al., 2021 (39)	?	+	?	?	?	?	?	+	+	+
Ma, et al., 2021 (41)	?	+	?	?	?	?	?	+	+	+
Noda, et al., 2021 (29)	?	+	?	?	?	?	?	+	+	+
Kuang, et al., 2020 (35)	?	+	?	?	?	?	?	+	+	+
Min, et al., 2020 (42)	+	+	?	?	?	?	?	+	+	+
Ma, et al., 2020 (40)	?	+	?	?	?	?	?	+	+	+
Chen, et al., 2019 (33)	?	+	?	?	?	?	+	+	+	+
Zhou, et al., 2019 (55)	?	+	?	?	?	?	?	+	+	+
Yu, et al., 2018 (51)	?	+	?	?	?	?	?	+	+	?
Hamet, et al., 2016 (31)	+	+	?	?	?	+	?	+	+	+
Li, et al., 2014 (38)	?	+	?	?	?	?	?	+	+	+

Green squares and plus sign (+) equate to “yes” meaning low bias, yellow squares and question mark (?) indicate “unclear,” and red with minus sign (−) indicate “no” meaning high risk for bias.

## 4 Discussion

The collective data from 28 *in vivo* manuscripts included in this systematic review provide convergent, thought not fully uniform, evidence supporting the bioactivity of microbial exopolysaccharides and their effects on intestinal health. Across a broad range of EPS doses (10–600 mg/kg), producing strains, and animal models, the included manuscripts demonstrated directionally similar effects, including enhanced colonic epithelial integrity, suppression of pro-inflammatory cytokine signaling, enrichment of beneficial commensal taxa, increased short-chain fatty acid production, and attenuation of oxidative stress. Notably, these effects were reported across EPS varying widely in producing taxon and, where reported, structural class which may point toward a degree of functional robustness shared across dietary polysaccharides. However, because structural and purity data were unavailable for nearly half of the included manuscripts (13 of 28), this apparent independence from structural classification cannot be formally tested with the current evidence base and should be treated as a preliminary observation rather than an established finding.

Additionally, the findings of this systematic review are consistent with and extend the existing literature linking dietary polysaccharides, as prebiotics, to gut homeostasis. The prebiotic activity of structural polysaccharides has long been mechanistically attributed to microbiome-mediated SCFA production (65–68), and the present systematic review corroborates this through consistent increases in acetate, propionate, and butyrate in fecal samples across included manuscripts spanning multiple EPS sources and animal models. However, the bioactive metabolite framework applied in this review investigating EPS is functionally distinct from prebiotic literature, as EPS were administered as isolated preparations rather than as components of intact fermented foods or probiotic co-cultures. Prior systematic reviews have examined postbiotics more broadly (69) or focused on EPS physicochemical properties and their functional roles in fermented food systems (70) rather than synthesizing *in vivo* colonic health outcomes specifically.

A terminological clarification is warranted given the ISAPP consensus definition of a postbiotic as “a preparation of a non-living microorganism and/or their components that confers a health benefit on the host” (71), a definition that explicitly excludes substantially purified metabolites in the absence of cellular biomass. Such compounds are instead to be named by establishing biochemical nomenclature. Under the postbiotic definition, the purified or highly enriched EPS preparations that compromised 17 of the 28 manuscripts in this review do not constitute themselves as postbiotics. Accordingly, we characterize EPS throughout this review as bioactive, fermentation-derived metabolites rather than postbiotics, and restrict any preparation-level postbiotic framing to studies using crude, biomass-retaining extracts. This distinction is not merely semantic: because purified EPS are typically administered as isolated, fermentable substrates rather than as components of a microbial preparation, several of the mechanisms discussed below (in particular, SCFA-mediated microbiome remodeling) are equally consistent with the ISAPP definition of a prebiotic (67). We do not attempt to adjudicate between postbiotic and prebiotic mechanistic frameworks for purified EPS in this review; rather, we flag this as an open classification question that the current characterized evidence base cannot resolve.

The intestinal effects of EPS that were documented across all included manuscripts in this systematic review are consistent with a proposed mechanistic axis in which EPS may contribute to microbiome remodeling, potentially leading to quantitative and qualitative modulation of the SCFA profiles, and ultimately resulting in epithelial barrier reinforcement and resolution of inflammation. This sequence is inferred from convergent associative findings across the included studies rather than demonstrated directly by any single manuscript. The frequently reported enrichment of *Akkermansia muciniphila* across multiple manuscripts (12/28) is particularly notable. *A. muciniphila* is a mucin-degrading symbiont whose abundance is inversely associated with intestinal permeability and colonic inflammation (72, 73); its promotion by structurally diverse EPS suggests that fermentable polysaccharide substrate availability, rather than EPS-specific receptor interactions, may be the primary driver of microbiome restructuring. Similarly, the frequently reported enrichment of the *Lactobacillaceae* and *Muribaculaceae* families, as well as the *Roseburia*, *Ruminococcus*, and *Blautia* genera aligns with expansion of saccharolytic, butyrogenic taxa. Butyrate, as the primary energy substrate for colonocytes, a histone deacetylase (HDAC) inhibitor, and a ligand for GPR109a and GPR41/43 on intestinal epithelial cells (74), offers a plausible mechanistic link between these microbiome shifts to the epithelial and immune outcomes reported in these manuscripts, though this pathway was not directly tested in the included studies.

At the epithelial level, EPS upregulated tight junction proteins ZO-1, OCLN, CLDN-1, and CLDN-5, alongside increased MUC2 secretion and goblet cell counts per cross section in the subset of manuscripts assessing each marker. This coordinated reinforcement of the mucosal barrier is mechanistically synergistic; claudin-1 regulates paracellular permeability, occludin modulates transcellular transport, ZO-1 anchors the tight junction complex to the actin cytoskeleton, and MUC2 forms the structural backbone of the colonic mucus layer (75). The parallel upregulation of these barrier components, together with histological restoration of crypt architecture, colon length, and gut barrier integrity, suggests that EPS may act upstream at the level of transcriptional regulation, potentially via butyrate-mediated HDAC inhibition or through modulation of pattern recognition receptor signaling, rather than through independent effects on individual structural proteins.

The anti-inflammatory signaling observed among the manuscripts that assessed these pathways converges on two well-characterized inflammatory cascades. First, EPS-mediated downregulation of TLR4 and MyD88 is consistent with reduced microbial pathogen-associated molecular pattern (PAMP) signaling, potentially reflecting the decreased (*p* < 0.05) relative abundance of *Escherichia*, a widely reported finding across included manuscripts, alongside a reduction in Proteobacteria reported in a single study, given that LPS is the canonical TLR4 ligand. Second, the consistent attenuation of NF-κB signaling (via decreased (*p* < 0.05) p65/p-p65 and IκBα phosphorylation) and MAPK signaling (including p38, JNK, and ERK pathways) would be expected to suppress transcription of TNF-α, IL-6, IL-1β, IFN-γ, IL-17, and IL-18, all of which decreased (*p* < 0.05) in EPS treatment groups, while promoting upregulation of IL-10 and TGF-β, consistent with a shift towards an immunoregulatory and tolerogenic immune response. The parallel activation of the Nrf2/Keap1 antioxidant pathway and the reduction in MPO activity reported across multiple manuscripts further indicate that EPS attenuate oxidative stress alongside inflammatory signaling. This is consistent with the well-established cross-regulation between oxidative and inflammatory cascades in the intestinal epithelium (76). Together, these findings support a model in which EPS act on the intestinal immune environment through a dual mechanism involving microbiome-dependent SCFA-mediated epigenetic and receptor signaling, as well as microbiome-dependent reduction of pro-inflammatory PAMP burden. The extent to which direct EPS-epithelial receptor interactions contribute independently of microbiome-mediated effects remains unclear and cannot be resolved from the current preclinical evidence base.

Several limitations affect the interpretation of this synthesis, spanning study-level methodology, the composition of the evidence base, and EPS characterization itself. Screening, full-text assessment, and data extraction were conducted by a single reviewer, which introduces risk of selection and extraction error. While the overall systematic review protocol and screening approach were discussed and agreed upon with a co-author prior to execution, formal independent dual-screening or a second-reviewer verification of extracted data was not performed; this is acknowledged as a limitation consistent with PRISMA recommendations for minimizing reviewer bias in future updates of this review. We sought to mitigate through broad, deliberately inclusive search terms and predefined PICOS criteria. This uncertainty is compounded by the underlying risk-of-bias profile of the primary literature: across the SYRCLE assessment, allocation concealment, random housing, and blinding (performance bias) were unclear in all 28 manuscripts, and sequence generation was unclear in more than half (15 of 28, 53.6%); only 2 of 28 manuscripts achieved low-risk ratings for random outcome assessment or blinding (detection bias). Given this pattern of predominantly unclear risk across core domains, the consistency of effect direction reported here should be interpreted as suggestive rather than definitive, and confidence in the synthesized findings is correspondingly limited.

Regarding the composition of evidence in this review, 25 of the 28 studies originated from China and the majority were published within the last several years. This likely reflects regional trends in EPS bioprospecting rather than the global evidence base and constrains the generalizability of these findings. The dominant animal model was DSS-induced murine colitis, which, while mechanistically informative, introduces model-specific pathophysiology that may not fully recapitulate human inflammatory bowel conditions; the limited representation of infection, cancer, and diet-induced dysbiosis models further constrains generalizability. The single zebrafish study included in this review (40) was retained as a validated intestinal and host-microbiome model despite its distinct route of exposure (1% m/m in feed) and phylogenetic distance from the predominantly mammalian evidence base, and its findings are interpreted as complementary to, rather than directly comparable with, the murine dose–response data reported elsewhere in this review. Additionally, the EPS dose range evaluated was substantial (10 to 600 mg/kg body weight). No dose–response relationship could be established due to most studies testing one or two doses with limiting evidence as to how the doses were selected which preludes a formal dose response analysis. Conflicting Firmicutes to Bacteroidetes (F/B) ratio outcomes across manuscripts, including both increases and decreases in each phylum depending on the study, highlight the limitations of this ratio as a univariate health index (77–80). These findings emphasize the need for functional, pathway-resolved microbiome analysis in future research.

Most consequential for the classification framework proposed above, EPS structural and purity characterization was inconsistent across the included manuscripts: 13 of 28 reported no structural or purity information at all, and among the remainder, some studies used well-defined purified compounds while others applied crude extracts from uncharacterized cultures or strains, with crude and purified preparations frequently pooled within the same synthesis. The apparent equivalence of outcomes across this structural and preparation-type diversity is biologically intriguing; however, it also means that structure–function attribution—and, by extension, confident postbiotic-versus-purified-metabolite classification at the level of individual studies—is not possible based on the current evidence base. This limits the rational design of EPS-based functional ingredients and means the terminological distinction drawn above should be read as a framework to guide future reporting, rather than a classification that can be retrospectively applied with confidence across all 28 included studies.

The evidence from this systematic review supports characterization of microbial EPS as bioactive metabolites acting on colonic health endpoints. This characterization should nonetheless be read as provisional and should not be extended uniformly to a postbiotic classification, given that (i) structural and purity information was unavailable for nearly half of the included manuscripts, (ii) crude and purified preparations were not evaluated separately for differential efficacy, and (iii) only preparations retaining microbial biomasses are consistent with the ISAPP postbiotic definition. Future primary research and reviews in this space would benefit from explicitly reporting and analyzing crude, biomass-retaining EPS preparations separately from purified EPS, consistent with established ISAPP terminology. This has practical significance for the food industry, where EPS are already produced at scale as fermentation byproducts and could be incorporated into functional food matrices, including fermented dairy products, plant-based beverages, and encapsulated supplements, pending confirmation of the intestinal health effects reported here in human trials, as the evidence synthesized in this review derives exclusively from preclinical animal models. The enrichment of *A. muciniphila*, butyrogenic *Lachnospiraceae* spp., and *Bifidobacterium* spp. observed across the included manuscripts is consistent with microbiome outcomes targeted by precision probiotic and prebiotic interventions in metabolic disease and inflammatory bowel disease contexts, suggesting mechanistic complementarity with existing gut health strategies.

## 5 Knowledge gaps, challenges, and future directions

This systematic review highlights the potential of EPS to support gut health; however, several important knowledge gaps and challenges remain. Most of the available research focuses on the colon, leaving the small intestine, a key site for digestion and nutrient absorption, largely uncharacterized. Next, although EPS consistently modulated gut microbiota composition, SCFA profiles, and intestinal barrier function, the underlying metabolic and molecular mechanisms remain poorly understood. In addition, there is considerable variation in the doses used across the included studies, making it difficult to define effective ranges for health-promoting outcomes associated with EPS in a food systems application. Furthermore, EPS characterization is inconsistent, as some studies investigated well-defined compounds, whereas others relied on crude extracts or uncharacterized strains. Interestingly, the majority of included manuscripts reported similar beneficial effects regardless of EPS microbial source, purity, or degree of chemical characterization, raising questions regarding the functional robustness of these compounds. Manuscripts that systematically investigate site-specific effects along the gastrointestinal tract, elucidate mechanistic pathways, standardize dosing strategies, and directly compare purified, well-characterized, and crude EPS preparations will be critical for translating preclinical findings into practical dietary interventions. Notably, structure–function relationships for EPS cannot be established from the current evidence base, as the majority of included studies did not report sufficient structural characterization. Future work explicitly designed to test structure–activity relationships is needed before EPS structural features can inform targeted dietary applications. Ultimately, translating these preclinical findings into dietary or functional food applications will require well-designed human clinical trials to confirm efficacy, establish safe and effective dosing in humans, and determine whether the barrier, immunomodulatory, and microbiome effects observed in murine models are recapitulated in the human gut.

## 6 Conclusion

This systematic literature review synthesizes the current body of evidence on the effects of microbial exopolysaccharides on intestinal health, highlighting a generally consistent association with modulation of the gut microbiota, alteration of the SCFA profile, and improvement of intestinal epithelial function. Collectively, these findings support the potential role of microbial exopolysaccharides as functionally relevant mediators of gut health. While the strength of conclusions is influenced by variability in experimental design and outcomes measured across studies, the overall evidence points to the biological relevance of EPS in host–microbe interactions. Together, this systematic review provides a consolidated framework for interpreting existing findings and underscores the need for continued, targeted research to advance mechanistic understanding and translational relevance.

## Footnotes

1
https://www.ncbi.nlm.nih.gov/pubmed

2
https://www.scopus.com

3
https://www.webofscience.com

4
https://www.webofscience.com/wos/fsta/basic-search
