# Supplementary material for: Microbial exopolysaccharides as bioactive modulators of intestinal health: a systematic review
Source: Front Nutr. 2026 Sep 7;13:1885879. doi: 10.3389/fnut.2026.1885879 (PMC13591379; doi:10.3389/fnut.2026.1885879)
Supplement: Supplementary file 1 [file Table_1.DOCX]

Supplementary Material

**Supplementary Table S1.** Search prepared according to the descriptors, in PubMed, Scopus, Web of Science, and Food Science and Technology Abstracts (FSTA).

| **Database** | **Descriptors** | **Items Found** | **Time** | **Date** |
| --- | --- | --- | --- | --- |
| **PubMed** | 1. "exopolysaccharide"[Title/Abstract] OR "EPS"[Title/Abstract] OR "postbiotic"[Title/Abstract] OR "probiotic metabolite" [Title/Abstract] | 23,820 | 14:51:39 | 12/10/25 |
|  | 2. "intestinal health"[Title/Abstract] OR "gut health"[Title/Abstract] OR "intestinal function"[Title/Abstract] OR "gut function"[Title/Abstract] OR "gut barrier"[Title/Abstract] OR "brush border membrane"[Title/Abstract] OR "intestinal epithelium"[Title/Abstract] OR "tight junction"[Title/Abstract] OR "zona occluden*"[Title/Abstract] OR "occludin"[Title/Abstract] OR "claudin"[Title/Abstract] OR "mucin"[Title/Abstract] OR "mucosa"[Title/Abstract] OR "mucus*"[Title/Abstract] OR "permeability"[Title/Abstract] OR "short chain fatty acid"[Title/Abstract] OR "SCFA*"[Title/Abstract] OR "butyrate*"[Title/Abstract] OR "propionate*"[Title/Abstract] OR "acetate*"[Title/Abstract] OR "gut microbiome"[Title/Abstract] OR "microbiome composition"[Title/Abstract] OR "microbial diversity"[Title/Abstract] OR "microbial community"[Title/Abstract] OR "microbiota"[Title/Abstract] OR "dysbiosis"[Title/Abstract] OR "inflammation"[Title/Abstract] OR "inflammatory response"[Title/Abstract] OR "immune response"[Title/Abstract] OR "cytokine*"[Title/Abstract] OR "TNF-alpha"[Title/Abstract] OR "oxidative stress"[Title/Abstract] OR "antioxidant"[Title/Abstract] OR "nutrient absorption"[Title/Abstract] OR "digestion"[Title/Abstract] | 2,594,449 | 14:52:27 |  |
|  | #1 + #2 | 4,658 | 14:52:38 |  |
| **Scopus** | 1. TITLE-ABS-KEY ( “exopolysaccharide” OR “EPS” OR “postbiotic” OR “probiotic metabolite” ) | 12,068 | 14:55 | 12/10/25 |
|  | 2. TITLE-ABS-KEY ( "intestinal health" OR "gut health" OR "intestinal function" OR "gut function" OR "gut barrier" OR "brush border membrane" OR "intestinal epithelium" OR "tight junction" OR "zona occluden*" OR "occludin" OR "claudin" OR "mucin" OR "mucosa" OR "mucus*" OR "permeability" OR "short chain fatty acid" OR "SCFA*" OR "butyrate*" OR "propionate*" OR "acetate*" OR "gut microbiome" OR "microbiome composition" OR "microbial diversity" OR "microbial community" OR "microbiota" OR "dysbiosis" OR "inflammation" OR "inflammatory response" OR "immune response" OR "cytokine*" OR "TNF-alpha" OR "oxidative stress" OR "antioxidant" OR "nutrient absorption" OR "digestion" ) | 4,959,218 | 14:56 |  |
|  | #1 + #2 | 10,237 | 14:57 |  |
| **Web of Science** | 1. TS=(“exopolysaccharide” OR “EPS” OR “postbiotic” OR “probiotic metabolite” ) | 40,450 | 14:58 | 12/10/25 |
|  | 2. TS=("intestinal health" OR "gut health" OR "intestinal function" OR "gut function" OR "gut barrier" OR "brush border membrane" OR "intestinal epithelium" OR "tight junction" OR "zona occluden*" OR "occludin" OR "claudin" OR "mucin" OR "mucosa" OR "mucus*" OR "permeability" OR "short chain fatty acid" OR "SCFA*" OR "butyrate*" OR "propionate*" OR "acetate*" OR "gut microbiome" OR "microbiome composition" OR "microbial diversity" OR "microbial community" OR "microbiota" OR "dysbiosis" OR "inflammation" OR "inflammatory response" OR "immune response" OR "cytokine*" OR "TNF-alpha" OR "oxidative stress" OR "antioxidant" OR "nutrient absorption" OR "digestion" ) | 3,753,383 | 14:58 |  |
|  | #1 + #2 | 12,235 | 14:59 |  |
| **FSTA** | 1. Topic search: "exopolysaccharide" OR "EPS" OR "postbiotic" OR "probiotic metabolite" | 4,823 | 15:00 | 12/10/25 |
|  | 2. Topic search: "intestinal health" OR "gut health" OR "intestinal function" OR "gut function" OR "gut barrier" OR "brush border membrane" OR "intestinal epithelium" OR "tight junction" OR "zona occluden*" OR "occludin" OR "claudin" OR "mucin" OR "mucosa" OR "mucus*" OR "permeability" OR "short chain fatty acid" OR "SCFA*" OR "butyrate*" OR "propionate*" OR "acetate*" OR "gut microbiome" OR "microbiome composition" OR "microbial diversity" OR "microbial community" OR "microbiota" OR "dysbiosis" OR "inflammation" OR "inflammatory response" OR "immune response" OR "cytokine*" OR "TNF-alpha" OR "oxidative stress" OR "antioxidant" OR "nutrient absorption" OR "digestion" | 318,623 | 15:01 |  |
|  | #1 + #2 | 1,556 | 15:02 |  |
